# O6-4 Passion, problems, and pathways forward: United Kingdom physical activity policy-makers' experiences of working in complex systems

**DOI:** 10.1093/eurpub/ckac094.044

**Published:** 2022-08-29

**Authors:** Benjamin Rigby, Caroline Dodd-Reynolds, Emily Oliver

**Affiliations:** 1 Department of Sociology, Durham University, Durham, United Kingdom; 2 Department of Sport and Exercise Sciences, Durham University, Durham, United Kingdom

**Keywords:** complexity, systems-thinking, policy-making, implementation, interviews

## Abstract

**Background:**

Insufficient attention to the inherent complexity of health ecosystems, through policy intervention, has precluded the development of sustainable and equitable solutions for health enhancing physical activity. While complexity and systems-thinking is becoming increasingly prevalent in policy discourse, little research has examined the implications of these approaches for developing national-level physical activity policies. Our project was the first to engage physical activity policy-makers, from across different sectors, in a discussion about their understanding of complexity and its effects on their efforts to influence systemic change.

**Methods:**

Ten national-level United Kingdom physical activity policy-makers were recruited through a purposive sampling strategy. Data was collected using semi-structured interviews. The data set was rigorously analysed using a thematic analysis.

**Results:**

All participants were bound by a collective culture of passion (theme 1) and enterprise in developing policy solutions for physical inactivity. However, making sense of the issue's complexity was not always easy. Our study revealed problems (theme 2) in that policy-makers were uncertain in their attempts to define complexity and its perceived implications for their work. Similarities drawn between physical inactivity and other public health issues resulted in policy-makers disassociating themselves from it, leaving them somewhat unable to identify their role and responsibility, and that of others, in addressing the problem. Pathways forward (theme 3) included leadership and other mechanisms to connect the system in effective advocacy, support and collaboration for physical activity promotion and policy implementation. Here connections were made to the ecological and technological developments that societies face.

**Conclusions:**

Our project advances knowledge on complexity and physical activity policy with critical implications for policy-making and broader decision-making for promoting health enhancing physical activity. The findings suggest that, in order to achieve reasonable and sustainable goals, it is important to embrace interconnectedness and create a shared platform for policy-makers to convene and reconcile their views and roles. Particular attention to identifying and operationalising leadership, policy levers and implementation factors in local systems is warranted. Focus needs to be placed on mechanisms that contribute to system cohesion, support the diffusion of best practice and knowledge exchange, and facilitate multi-sectoral policy-making.

